# A carbon nanotube-modified electrode for a highly active and reversible Sn^4+^/Sn anode

**DOI:** 10.1039/d5sc08606j

**Published:** 2026-01-19

**Authors:** Yue Ao, Yonggang Wang, Shuo Wang, Chengji Zhao, Congxin Xie, Xianfeng Li

**Affiliations:** a Key Laboratory of High Performance Plastics, Ministry of Education, College of Chemistry, Jilin University Changchun 130012 China zhaochengji@jlu.edu.cn; b Division of Energy Storage, Dalian Institute of Chemical Physics, Chinese Academy of Sciences Dalian 116023 China xiecongxin@dicp.ac.cn lixianfeng@dicp.ac.cn; c Department of Chemistry, Shanghai Key Laboratory of Molecular Catalysis and Innovative Materials, Institute of New Energy, iChEM (Collaborative Innovation Center of Chemistry for Energy Materials), Fudan University Shanghai 200433 China

## Abstract

Tin (Sn) is an attractive anode for high energy density batteries due to its four-electron redox process (Sn^4+^ → Sn^2+^ → Sn) without dendrite formation. However, the sluggish kinetics and poor reversibility of the Sn^4+^/Sn^2+^ process hinder its practical implementation. Herein, we propose a surface-engineering strategy to accelerate the Sn^4+^/Sn^2+^ redox kinetics and enable highly reversible Sn^4+^/Sn reactions. Specifically, carbon nanotubes (CNTs) enriched with edge defects and oxygen-containing groups are grown *in situ* on carbon felt (CF) *via* chemical vapor deposition (CVD), forming a high surface area electrode (denoted as CC-*T*). These CNTs provide abundant active sites for Sn^4+^ adsorption and facilitate charge transport, thereby enhancing electron transfer kinetics and redox reversibility. Consequently, the charge-transfer resistance (*R*_ct_) of CC-*T* decreased by more than 55-fold compared with pristine CF (0.27 *vs.* 14.89 Ω). When assembled in a Sn/Br flow battery, the battery delivered an energy efficiency (EE) of 80% at 40 mA cm^−2^, outperforming that of pristine CF (63%), and maintaining stable cycling for over 650 hours. Even with 4 M electrolyte, the battery achieved a discharge capacity of 373 Ah L^−1^ and an areal capacity of 614 mAh cm^−2^. This work provides a promising approach for developing high-capacity, dendrite-free metal anodes for next-generation flow batteries.

## Abstract 2

*In situ*-grown CNTs decorated on carbon felts overcome reversibility issues of Sn^4+^/Sn couple, enabling a Sn/Br flow battery with 80% energy efficiency at 40 mA cm^−2^, 650 h stability, 373 Ah L^−1^ discharge capacity and 614 mAh cm^−2^ areal capacity.

## Graphical abstract

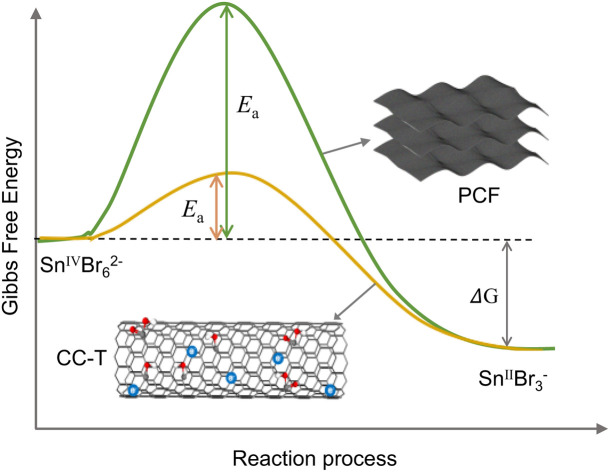

## Introduction

Aqueous flow batteries (AFBs) are promising in energy storage due to their ability to decouple power and energy, and their long cycle life and excellent safety.^1–5^ However, their energy density is lower than that of power-dense technologies such as lithium-ion batteries.^6,7^ Flow batteries can be divided into traditional and hybrid systems. Traditional systems, such as vanadium flow batteries (*e.g.*, V^2+^/V^3+^), rely on liquid-to-liquid electrochemical reactions,^8^ while hybrid systems typically use deposition/dissolution-based negative electrodes, such as Zn^2+^/Zn, which offer higher specific capacities (*e.g.*, Zn^2+^/Zn at 820 mAh g^−1^ or >268 Ah L^−1^) and higher energy densities (*e.g.*, the theoretical energy density of zinc–bromine flow batteries exceeds 400 Wh kg^−1^).^9,10^ This is attributed to the high solubility of metal ions (>5 M for Zn^2+^) and the multi-electron transfer reaction in the deposition/dissolution process. Despite extensive studies on zinc anodes in aqueous flow batteries, challenges such as dendrite growth and water corrosion continue to limit their cycle life and areal capacity (<50 mAh cm^−2^).^11–13^

Compared to zinc (−0.76 V),^14^ the redox potential of tin is higher (−0.15 V), addressing water corrosion and hydrogen evolution.^15^ In addition, its body-centered tetragonal crystal structure ensures isotropic surface energy during deposition, preventing dendrite formation.^16,17^ Because of these properties, tin is an ideal material for metallic anodes, leading to the development of Sn^2+^/Sn-based batteries,^18,19^ such as tin–iron,^20^ tin–bromine,^21^ and tin–manganese^22^ flow batteries, which offer high power density and cycle stability. However, tin also exists in the higher oxidation state of Sn^4+^,^23,24^ and if the four-electron transfer process of Sn^4+^/Sn could be realized, coupled with the high solubility of tin salts (*e.g.*, SnCl_4_ > 4 M), the energy density of tin-based batteries could be significantly enhanced.

Currently, the reduction of Sn^4+^ to Sn^2+^ faces significant challenges,^15^ primarily due to substantial electrochemical polarization and poor reversibility, which results in this reaction being typically considered electrochemically inert.^18^ This is because Sn^4+^ is a small ion with a high charge density, and readily forms strong complexes with anions (such as Cl^−^ or Br^−^) or water molecules,^25–27^ which significantly hinders the electron transfer process. Additionally, because of the stable 4d^10^ electron configuration in the outer shell of Sn^4+^, gaining electrons is difficult.^28^ Therefore, the key to advancing high-performance four-electron transfer (Sn^4+^/Sn) tin-based flow batteries lies in achieving fast and reversible Sn^4+^/Sn^2+^ reactions:

1 Sn^4+^ + 2e^−^ ⇌ Sn^2+^; *E*^0^ = 0.15 V

2 Sn^2+^ + 2e^−^ ⇌ Sn; *E*^0^ = −0.14 V

Electrode interface modification is one of the core strategies for enhancing electrochemical performance. Carbon nanomaterials, with their high specific surface area (80–200 m^2^ g^−1^) and abundant functional groups (*e.g.*, –COOH, –OH), offer significant advantages in increasing electrochemical reaction carbon defects and oxygen-containing functional groups.^31^ These characteristics not only optimize the adsorption of Sn^4+^, but also accelerate interfacial charge transfer, thereby holding great potential for significantly improving the kinetics and reversibility of the Sn^4+^/Sn^2+^ reaction.

Herein, we report a highly active electrode capable of achieving a reversible and fast Sn^4+^/Sn^2+^ reaction. Using chemical vapor deposition (CVD), carbon nanotubes (CNTs) can be grown *in situ* on carbon felt (CF) electrodes (denoted as CC-*T*). The CC-*T*, with abundant active functional groups and defect sites, enhances Sn^4+^ adsorption and interfacial reaction kinetics. Cyclic voltammetry (CV) shows a reduced peak potential difference from 410 mV to 240 mV, improving battery reversibility, while electrochemical impedance drops by an order of magnitude.

Full-cell tests with a Br_2_/Br^−^ cathode show that the energy efficiency (EE) increased from 63% (pristine CF) to 80% at 40 mA cm^−2^, with stable operation exceeding 650 hours. The specific capacity of Sn reached 887 mAh g^−1^ (based on the anode), with an electrolyte utilization of 98%. Additionally, benefiting from the high solubility of Sn^4+^ and the four-electron transfer process, the discharge capacity increased to 373 Ah L^−1^ with over 860 hours of operation. More importantly, due to the absence of dendrite growth, the areal capacity of the anode exceeded 614 mAh cm^−2^, providing a solid foundation for the future development of high-capacity anodes.

## Results and discussion

### Electrode preparation and characterization analysis

**Fig. 1:**
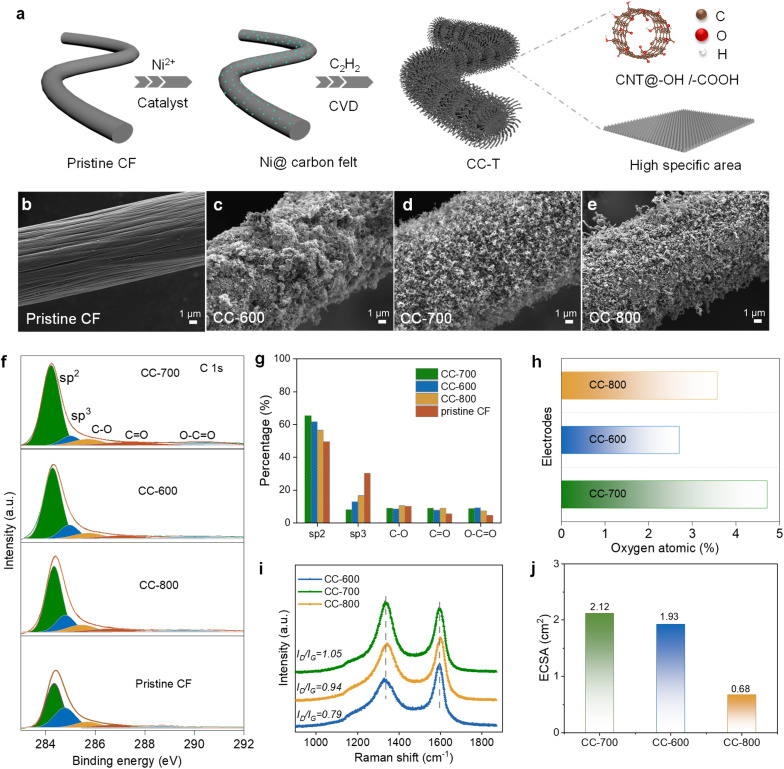
Characterization and analysis of the prepared electrodes. (a) A schematic illustration of the fabrication process for high-performance electrodes. A high specific surface area and abundant surface functional groups (*e.g.*, carboxyl groups: –COOH, and hydroxyl groups: –OH) contribute to the enhancement of the reaction kinetics. (b–e) SEM images of a series of comparative electrodes: (b) pristine CF, (c) CC-600, (d) CC-700, and (e) CC-800. (f) XPS analysis of C 1s peaks for the different electrodes. (g) Ratios of functional groups calculated from the XPS results in (f). (h) A comparison of the oxygen content of different electrodes. (i) Raman spectra of the different electrodes. (j) A comparison of the ECSA of the different electrodes. The ECSA (cm^2^) value was determined according to the equation^36^ ECSA = *C*_dl_/*C*_s_, where *C*_dl_ was derived from Fig. S8, and *C*_s_ = 80 µF cm^−2^ is the specific value of the capacitance on a porous, highly active, and inhomogeneous surface.^37^

The preparation of high-performance electrodes involves a facile process: first, pristine CF is immersed in a Ni^2+^ solution and dried. In the subsequent CVD process, the nickel source serves as a catalyst; acetylene (C_2_H_2_) gas is introduced to the Ni surface under different temperatures (600–800 °C), enabling the *in situ* growth of CNTs on CF (CC-*T*)^29^ (Fig. 1a). Scanning electron microscopy (SEM) images and corresponding energy-dispersive X-ray spectroscopy (EDS) mappings (Fig. S1) revealed that the nickel was uniformly distributed across the CF surface. Compared to pristine CF, the CNTs formed by the CVD method uniformly cover the surface of the CF, significantly increasing the specific surface area (Fig. 1b–e).^30^

To optimize the electrode structure, CNTs were deposited at varying temperatures (600–800 °C). Among these, the sample prepared at 700 °C exhibited the most uniform morphology (Fig. S2) and the highest specific surface area (223 m^2^ g^−1^), outperforming those prepared at 600 °C (197 m^2^ g^−1^) and 800 °C (162 m^2^ g^−1^) (Fig. S3). Following deposition, acid etching was carried out to remove the metallic nickel, which effectively eliminated the hydrogen evolution reaction (HER). X-ray diffraction (XRD) and transmission electron microscopy (TEM) analyses confirmed the complete removal of nickel (Fig. S4a and b), while the morphology of the CC-*T* remained largely intact post-etching (Fig. S4c).

X-ray photoelectron spectroscopy (XPS) was employed to investigate the effects of the functional groups and their contents on the CC-*T*. The deconvolution of the XPS spectra (Fig. 1f) revealed differences in the concentrations of oxygen-containing functional groups and sp^2^-hybridized carbon in electrodes prepared under different temperature conditions. Fig. 1g and h illustrates the ratios of functional groups, and it can be concluded that CC-700 possesses the highest concentration of oxygen-containing functional groups. This specific composition significantly enhanced the local interfacial charge density, thereby facilitating the electro-transfer process.

Fourier-transform infrared (FTIR) analysis further illustrates the specific adsorption between Sn^4+^ and oxygen-containing functional groups. When CC-700 was treated with Sn^4+^ solution, the C=O peak at 1646 cm^−1^ obviously diminished,^32^ while the intensity of the C–O peak at 1084 cm^−1^ significantly decreased due to coordination with Sn ions,^33^ and then, a new Sn–O peak emerged at 676 cm^−1^ (ref. 34) (Fig. S5). To evaluate the capacity for Sn^4+^ adsorption by different electrodes, inductively coupled plasma optical emission spectrometry (ICP-OES) tests were conducted on pristine CF and pre-oxidized CF. The results indicated that the electrode with the higher oxygen content exhibited stronger adsorption of Sn^4+^ (Fig. S6).

Raman spectroscopy was utilized to assess the defect density within the carbon materials. The D-band to G-band intensity ratio (*I*_D_/*I*_G_) in CC-700 is 1.05, higher than CC-800 (0.94) or CC-600 (0.79), which indicates an increased number of defects within the carbon structure (Fig. 1i). Additionally, TEM was employed to examine the stacking behavior of the crystal planes in the carbon materials (Fig. S7). For the CNTs deposited at 800 °C, discontinuities and collapse in the crystal structure were observed. At 600 °C, the relatively low deposition temperature resulted in poorly defined crystal plane arrangements and significant structural disorder. In contrast, the sample synthesized at 700 °C demonstrated distinct CNT crystal plane spacing and a regular arrangement, and this ordered structure can reduce electron transmission resistance.^35^

The electrochemical active surface areas (ECSAs) were quantitatively assessed through CV tests. As shown in Fig. S8d, the slope value of the current-scan rate of the CC-700 is significantly higher than that of the other electrodes, with the largest ECSA shown in Fig. 1j. These results demonstrate that the most active sites are on CC-700, and this trend is consistent with the aforementioned spectroscopic analysis.

### Adsorption mechanism analysis of Sn^4+^/Sn^2+^ coordination ions on the surface of CNTs

**Fig. 2:**
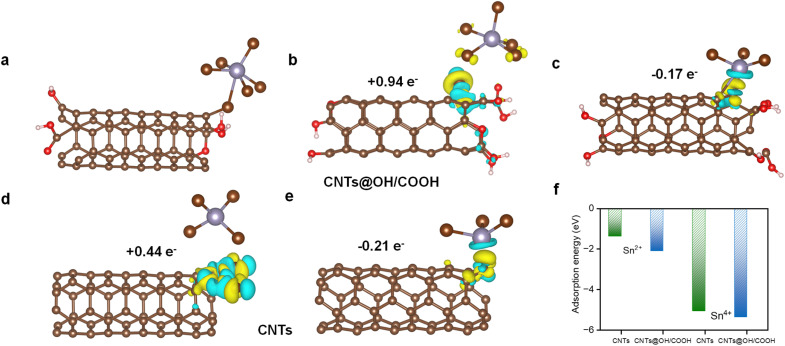
Adsorption characteristics of SnBr_6_^2−^/SnBr_3_^−^ on CNTs or CNTs@OH/COOH. (a) Adsorption model diagrams for Sn coordination ions on CNTs@OH/COOH. The charge density difference diagram is shown in (b–e) for (b) SnBr_6_^2−^ and (c) SnBr_3_^−^ ions adsorbed on CNTs@OH/COOH, and (d) SnBr_6_^2−^ and (e) SnBr_3_^−^ ions adsorbed on CNTs. The yellow parts represent regions with electron localization, while the blue parts represent electron delocalization. The Bader charge was calculated *via* electron cloud computation. (f) Adsorption energy simulations of SnBr_6_^2−^ and SnBr_3_^−^ on CNTs and CNTs@OH/COOH, respectively.

Tin ions with high positive charge density tend to form stable coordination structures with halogen anions, such as Br^−^ and Sn^4+^, and typically coordinate with six Br^−^ (SnBr_6_^2−^), while the coordination number of Sn^2+^ is three (SnBr_3_^−^).^25–27^ The Material Studio (MS) program was applied to constructed CNT models to analyze the ion adsorption behavior. Electronic analysis revealed that a strong electronic interaction formed between Br^−^ in SnBr_6_^2−^ and the CNTs containing –OH and –COOH (denoted as CNTs@OH/COOH) (Fig. 2a).

This interaction induced the Br^−^ to directly dissociate from the coordination environment and chemisorb onto the CNTs@OH/COOH surface. After stabilization, electrons were transferred from the CNTs@OH/COOH to SnBr_6_^2−^, and thus, SnBr_6_^2−^ gained a total of 0.94e^−^ (Fig. 2b). This result indicates a stable electronic coupling interaction between metal ions and electrode surface. Next, the adsorption strength between SnBr_3_^−^ and CNTs@OH/COOH weakened, which occurred because the central Sn atom hybridized with the CNTs@OH/COOH, and 0.17e^−^ were transferred from SnBr_3_^−^ to the CNTs@OH/COOH (Fig. 2c). This result demonstrates the reducibility of the stannous ion (Sn^2+^).

Similarly, Fig. 2d and e shows the adsorption of SnBr_6_^2−^ and SnBr_3_^−^ on the surface of CNTs without oxygen-functional group modification. Compared with CNTs@OH/COOH, the overall adsorption capacity of CNTs is weaker, and their electron-accepting ability is also weaker than CNTs@OH/COOH (Bader charge: 0.94 *vs.* 0.44*e*^−^ and −0.17 *vs.* −0.21*e*^−^). In Fig. 2f, the curve shows that the adsorption energy of SnBr_3_^−^ is much lower than that of SnBr_6_^2−^, and when CNTs are used as the substrate, the adsorption strength of SnBr_6_^2−^ and SnBr_3_^−^ decreases as compared to that of CNTs@OH/COOH. This indicates that the presence of oxygen-containing functional groups is beneficial for the adsorption of Sn coordination ions, and it promotes the occurrence of subsequent charge transfer.

The desorption energy for the final Sn product (after removing all Br^−^ ligands) was −0.45 eV on CNT@OH/COOH, *versus* 0.59 eV on CNTs (Fig. S9). This indicates that the presence of oxygen-containing functional groups promotes the desorption of Sn, which facilitates the release of active sites and further validates the correlation that a higher density of oxygen-containing functional groups represents a larger ECSA.

### Electrochemical kinetics characterization of different electrodes

**Fig. 3:**
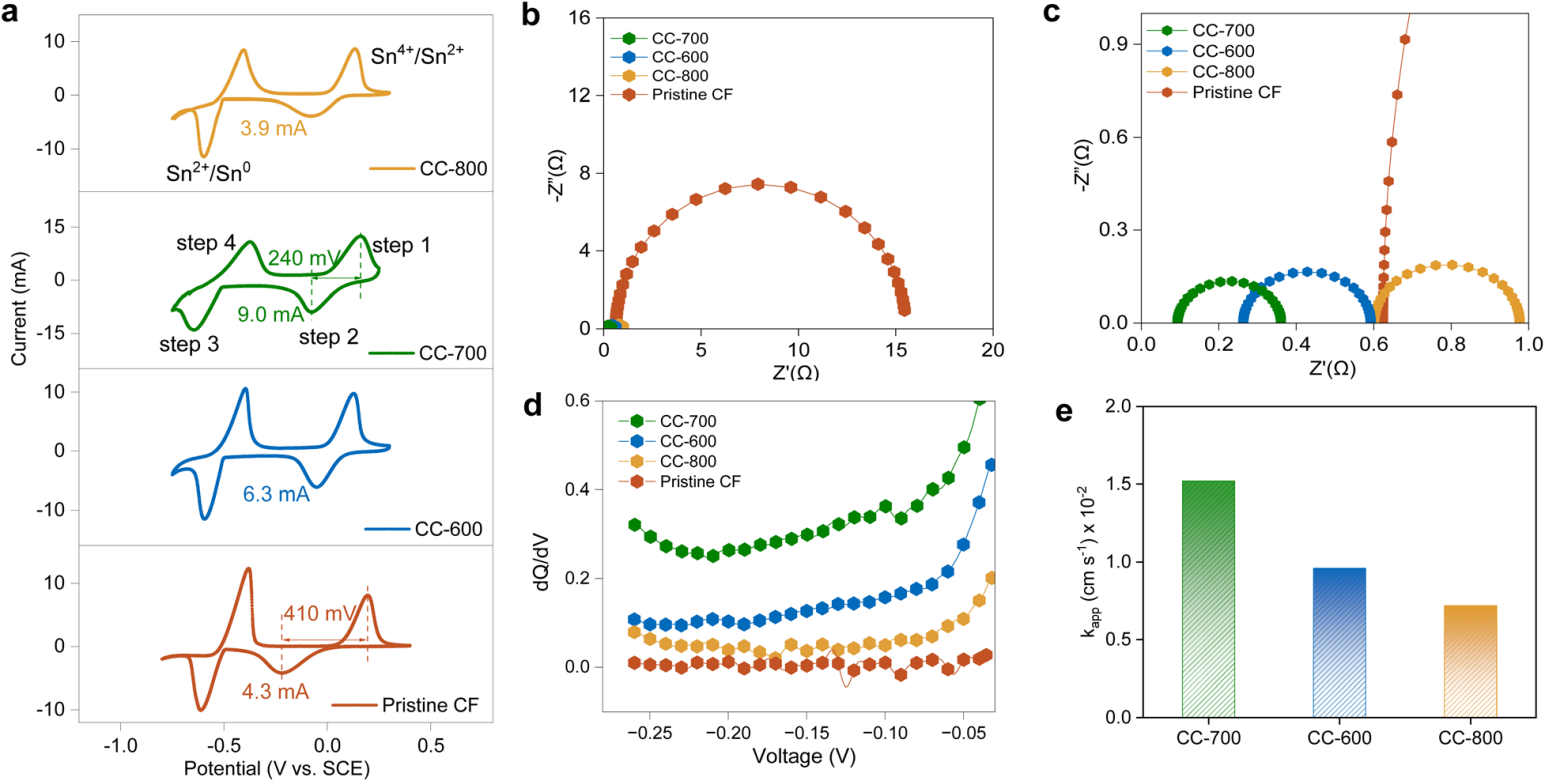
Electrochemical performance characterization of CC-*T*. (a) CVs of 20 mM SnCl_4_ + 2 M HBr with working electrodes as follows: yellow: CC-800, green: CC-700, blue: CC-600, and red: pristine CF, at a scan rate of 5 mV s^−1^. (b and c) Electrochemical impedance spectroscopy (EIS) results revealing that the CC-700 electrode exhibits the lowest charge transfer resistance (*R*_ct_) for the Sn^4+^/Sn^2+^ redox couple. (d) Differential capacity curves of the different electrodes, derived from the CV data in (a). CC-700 shows a higher d*Q*/d*V* compared to the other electrodes, indicating its superior charge-storage capability. (e) Apparent rate coefficient *K*_app_ (cm s^−1^) for the Sn^4+^ → Sn^2+^ reduction reaction on various electrodes, which was calculated from Fig. S11, S12 and Table S1.

To evaluate the electrochemical reversibility of the Sn^4+^/Sn^2+^ redox process, CV measurements were conducted (Fig. 3a), primarily focusing on the redox behavior of step (2) (Sn^4+^ → Sn^2+^). The pristine CF electrode exhibited poor electrochemical reversibility, while the CC-700 electrode significantly enhanced the reversibility, with the peak separation of Sn^4+^/Sn^2+^ decreasing from 410 mV to 240 mV. Furthermore, CC-700 exhibited the highest reduction peak current (9 mA), significantly outperforming the pristine CF and other CC-*T*s.

Electrochemical impedance spectroscopy (EIS) was employed to analyze the charge transfer resistance (*R*_ct_), a parameter inversely related to the electron transfer rate. As shown in Fig. 3b, c and S10, the CC-700 electrode exhibits the smallest *R*_ct_ (0.27 Ω), approximately 1/55th of the *R*_ct_ of the pristine CF electrode (14.89 Ω). Differential capacitance curves (d*Q*/d*V*), derived from the CV profiles in Fig. 3a, were used to assess the electrode's charge storage capacity. During the range of −0.25 to −0.05 V, no electrochemical reactions occur. Only the charge–discharge behavior of the electric double layer occurs, which consists of active ions diffusing and accumulating toward the surface of the electrode. Quantitative analysis revealed that the d*Q*/d*V* value of the CC-700 electrode is higher when compared to that of the other electrodes (Fig. 3d). This confirms its superior active ion storage ability, and provides a mass transport foundation for the subsequent reaction.

Among the electrodes, CC-700 exhibited the highest ECSA. After normalizing the intrinsic rate constant *k*_0_ by ECSA, CC-800 demonstrated the highest per-site conversion efficiency (CE), as summarized in Table S1. However, the evaluation of battery performance is normalized by geometric area, such as current density (mA cm^−2^), and thus, employing the apparent rate constant *K*_app_ is more consistent.^16,17^ As shown in Fig. 3e, the *K*_app_ value for CC-700 is 1.52 × 10^−2^ cm s^−1^, which is significantly higher than that for other electrodes, and indicates its superior performance. This suggests that the performance enhancement primarily results from the catalytic effect and the increased active surface area of CC-700.

From the perspective of overall battery performance, CC-700 is more suitable for application. Additionally, the apparent diffusion coefficients (*D*_app_) for Sn^4+^ → Sn^2+^ are also determined from the CV measurements conducted at varying scan rates. The CC-700 electrode demonstrated a significantly superior *D*_app_ value of 13.69 × 10^−6^ cm^2^ s^−1^, which was higher than that of CC-600 (12.15 × 10^−6^ cm^2^ s^−1^), CC-800 (8.17 × 10^−6^ cm^2^ s^−1^), and pristine CF (6.09 × 10^−6^ cm^2^ s^−1^) (see Fig. S11, S13 and Table S2 for details).

### High-performance Sn-based flow batteries

**Fig. 4:**
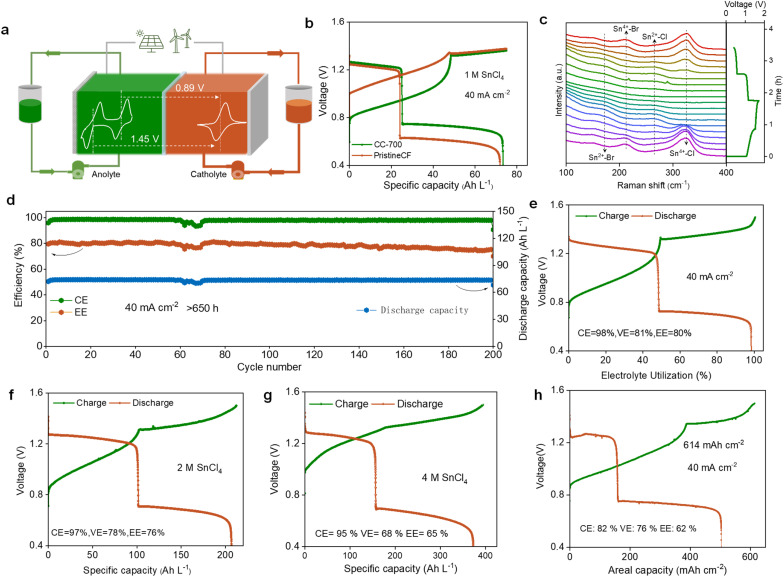
The battery performance of Sn/Br flow batteries utilizing CC-700 demonstrating enhanced efficiency and stability. (a) A schematic diagram of the assembled Sn/Br redox flow battery (RFB). The inset shows CV curves of 20 mM SnCl_4_ + 2 M HBr (left line) and 20 mM HBr + 2 M H_2_SO_4_ (right line), respectively, scanned at a rate of 5 mV s^−1^. (b) Charge–discharge curves of Sn/Br ARFBs. Electrolyte: 1 M SnCl_4_ + 0.16 M choline chloride (ChCl) + 2 M HBr. Choline chloride, as a complexing agent for Br_2_, was employed to reduce the corrosive effects of Br_2_. The polarization of the voltage curve for CC-700 is significantly lower than that for pristine CF. (c) *In situ* Raman tests during the charge–discharge process of Sn/Br flow batteries. The coordination structures of Sn^4+/2+^–Br and Sn^4+/2+^–Cl showed reversible change processes. (d) Long-term cycle stability testing. Electrolyte: 1 M SnCl_4_ + 0.16 M ChCl + 2 M HBr. The battery operated stably for over 650 hours (or 200 cycles) at a current density of 40 mA cm^−2^. (e) The charge–discharge curve of high specific capacity, with electrolyte utilization reaching 98%. Electrolyte: 1 M SnCl_4_ + 2 M HBr + 0.16 M ChCl. (f) Charge–discharge voltage profiles of the battery with 2 M Sn^4+^ as the anolyte. Anolyte: 2 M SnCl_4_ + 0.16 M ChCl + 2 M HBr; catholyte: 1 M SnCl_4_ + 0.16 M ChCl + 2 M HBr. The discharge specific capacity reached 207 Ah L^−1^. (g) Charge–discharge profiles with 4 M Sn^4+^ as the anolyte. Anolyte: 4 M SnCl_4_ + 0.16 M ChCl + 2 M HBr; catholyte: 1 M SnCl_4_ + 0.16 M ChCl + 2 M HBr. The discharge specific capacity reached 373 Ah L^−1^. (h) Battery performance testing under high areal capacity conditions (>614 mAh cm^−2^). Electrolyte: 1 M SnCl_4_ + 0.16 M ChCl + 2 M HBr.

In this study, the electrolyte composition consists of SnCl_4_ + HBr + ChCl, where HBr not only serves as the supporting electrolyte but also provides Br^−^ as the redox species for the cathode, while choline chloride (ChCl) acts as a complexing agent for Br_2_. Compared to traditional metal anodes (*e.g.*, Zn^2+^/Zn), the tin anode offers several advantages, including multiple electron transfers (Sn^4+^/Sn), higher solubility (>4 M), and the absence of dendrite growth. The anodic couples of Sn^4+^/Sn^2+^ and Sn^2+^/Sn, together with the cathodic couple Br^−^/Br_2_, exhibited open-circuit voltages (OCVs) of 1.45 V and 0.89 V, respectively (Fig. 4a). As illustrated in Fig. 4b, the Sn/Br battery exhibited two distinct charge–discharge plateaus, corresponding to the Sn^4+^/Sn^2+^ and Sn^2+^/Sn redox reactions. With CC-700, the battery achieved an EE of 80%, which is significantly higher than the 63% obtained with the pristine CF (Fig. 4b and S14).

*In situ* Raman spectroscopy was employed to confirm the electrochemical mechanism. During the first step of charging, the signal intensities of Sn^4+^–Cl (326 cm^−1^) and Sn^4+^–Br (213 cm^−1^) gradually weakened and eventually disappeared, and Sn^2+^–Cl and Sn^2+^–Br signals emerged. During the second charging step, these Sn^2+^–Cl and Sn^2+^–Br signals gradually faded, indicating the deposition of metallic Sn from Sn^2+^. The discharge process displayed the reverse sequence, corroborating the CV test results (Fig. 4c and S15). The SEM images show that the tin layer exhibits a smooth and compact morphology, demonstrating good application potential (Fig. S16). The battery, assembled with 1 M SnCl_4_, demonstrated stable operation over 200 cycles with negligible loss in efficiency and capacity, achieving a CE of 98% and an EE of 80% (Fig. 4d and S17). After 200 cycles, tin continued to maintain uniform deposition, and the cross-sectional SEM images showed no dendritic growth (Fig. S18).

In addition, SEM analysis revealed that the morphological changes in CC-700 after cycling were minimal (Fig. S19). Raman analysis showed a slight increase in the *I*_D_/*I*_G_ ratio, which may be due to the etching effect in the acidic electrolyte, leading to the formation of additional defects (Fig. S20). XPS analysis further indicated that the oxygen content slightly increased after cycling, but the oxygen-containing functional groups remained nearly unchanged (Fig. S21). These results fully demonstrate the excellent stability of CC-700 during long-term cycling.

Due to the Sn dendrite-free property and excellent stability of CC-700, the utilization ratio of the anolyte reached 98%, and the battery stably operated for over 248 hours (Fig. 4e and S22). The performance of the electrodes at different current densities was also explored. For the CC-700 electrode, 98% CE was attained at 40 mA cm^−2^, which increased to 99% at 120 mA cm^−2^. Additionally, the voltage efficiency (VE) of the battery incorporating the CC-700 electrode sustained 80% at 40 mA cm^−2^, and remained above 63% even at 120 mA cm^−2^. This performance is notably superior to the pristine CF electrode-based battery, which merely attained 65% at 40 mA cm^−2^ and 51% at 120 mA cm^−2^. Furthermore, the CC-700 electrode also outperformed other fabricated electrodes (CC-600 and CC-800) in efficiency, which is consistent with the CV results. The excellent performance of the CC-700 electrode is mainly attributed to its ability to activate the Sn^4+^/Sn^2+^ redox reaction and reduce battery polarization (Fig. S23 and S24).

Increasing the SnCl_4_ concentration to 2 M resulted in a discharge specific capacity of 207 Ah L^−1^ (196 Wh L^−1^), with stable operation for more than 340 hours (Fig. 4f and S25). When the concentration was further increased to 4 M, the discharge-specific capacity rose to 373 Ah L^−1^ (333 Wh L^−1^), with stable operation for 860 hours (Fig. 4g and S26). Moreover, a high areal capacity of 614 mAh cm^−2^ (Fig. 4h) was realized with a charging time exceeding 15 hours (Fig. S27). Even at such a high areal capacity, the deposited tin still exhibited no obvious dendrite formation (Fig. S28).

### Opportunity and challenge for Sn-based AFBs

**Fig. 5:**
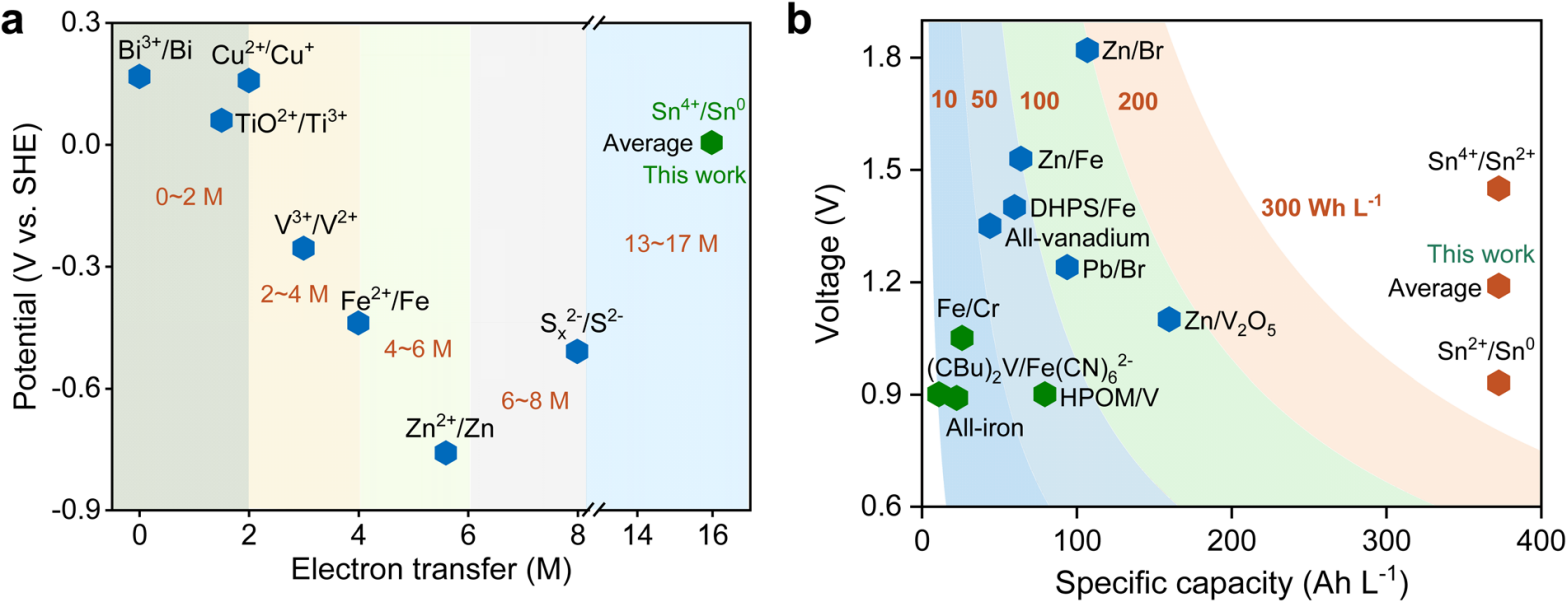
Compared to other reported redox systems, Sn^4+^/Sn demonstrates a superior electron transfer number and specific capacity. The electron transfer number and redox potential in (a) are obtained from the following references: Zn^2+^/Zn,^38^ Bi^3+^/Bi,^39^ Fe^2+^/Fe,^40^ Cu^2+^/Cu^+^,^41^ TiO^2+^/Ti^3+^,^42^ V^3+^/V^2+^,^43^ and S_*x*_^2−^/S^2−^.^44^ The specific capacity and battery voltage in (b) are obtained from the following references: Zn/V_2_O_5_,^45^ all-vanadium,^46^ all-iron,^47^ Fe/Cr,^48^ Zn/Br,^49^ Zn/Fe,^50^ Pb/Br,^51^ DHPS/Fe,^52^ HPOM/V,^53^ and (CBu)_2_V/[Fe(CN)_6_]^2−^.^54^

Compared to other conventional anode materials, acidic SnCl_4_ stands out with a remarkable solubility exceeding 4 M, an inherent electron transfer number of 4, and a theoretically achievable electron transfer capacity of 16 M—substantially surpassing that of traditional anodes (Fig. 5a). In the assembled Sn/Br flow battery system, the specific capacity reached 373 Ah L^−1^, significantly outperforming other reported aqueous battery systems (Fig. 5b). Therefore, because of the high solubility of Sn^4+^, its four-electron transfer capability, and the absence of dendrite growth, tin-based batteries exhibit numerous benefits in terms of energy density and cycling life. These results further confirm the significant advantages of Sn-based anodes for flow battery applications.

## Conclusions

By leveraging a CNT-modified CF electrode (CC-*T*) through CVD, we significantly enhanced the electrochemical kinetics and reversibility of the Sn^4+^/Sn^2+^ system. This modification not only increased the specific surface area, but also introduced a wealth of active sites, particularly those enriched with edge defects and oxygen groups. These structural improvements facilitated superior Sn^4+^ adsorption and accelerated charge transport, resulting in a remarkable reduction in electrochemical transfer impedance to just 1/55 of that observed in pristine CF. Additionally, the redox peak potential difference in HBr electrolyte was significantly narrowed from 0.41 V to 0.24 V.

The Sn/Br flow battery, featuring a Br^−^/Br_2_ cathode, achieved an impressive EE of 80% at 40 mA cm^−2^, outperforming the pristine CF electrode (63%) and demonstrating stable performance over 650 hours. When operated with 4 M SnCl_4_ electrolyte, the battery showcases a high discharge capacity of 373 Ah L^−1^ and an outstanding areal capacity of 614 mAh cm^−2^, all while maintaining a dendrite-free morphology throughout its operation. These promising results underscore the substantial potential of Sn-based anodes for high-performance aqueous flow batteries, setting the stage for their broader application in future energy storage systems that offer enhanced capacity, efficiency, and long-term stability.

## Author contributions

Y. A. carried out experiments and wrote the manuscript. S. W. performed the calculations. Y. G. W. and C. J. Z. revised the manuscript. C. X. X. and X. F. L. supervised the project and provided funding support.

## Conflicts of interest

There are no conflicts to declare.

## Supplementary Material

SC-OLF-D5SC08606J-s001

## Data Availability

The data supporting this article are available within the main text and the supplementary information (SI). Supplementary information: materials, methods and supplementary data. See DOI: https://doi.org/10.1039/d5sc08606j.
