# Supplementary material for: A carbon nanotube-modified electrode for a highly active and reversible Sn4+/Sn anode
Source: Chem Sci. 2026 Jan 19;17(13):6448–55. doi: 10.1039/d5sc08606j (PMC12875277; doi:10.1039/d5sc08606j)
Supplement: SC-017-D5SC08606J-s001 [file SC-017-D5SC08606J-s001.pdf]

Supplementary Information (SI) for Chemical Science.
This journal is © The Royal Society of Chemistry 2026

Supplementary Information

Carbon Nanotube-Modified Electrode for a Highly Active and Reversible Sn^{4+}/Sn Anode

Yue Ao [a,b], Yonggang Wang [c], Shuo Wang [b], Chengji Zhao *[a], Congxin Xie *[b] and Xianfeng Li *[b]

a Y. Ao, Prof. C. J. Zhao
Key Laboratory of High Performance Plastics Ministry of Education College of Chemistry, Jilin University Changchun 130012, China
E-mail: zhaochengji@jlu.edu.cn

b Y. Ao, Dr. S. Wang, Dr. C. X. Xie, Prof. X. F. Li
Division of Energy Storage Dalian Institute of Chemical Physics Chinese Academy of Sciences Dalian 116023, China
E-mail: xiecongxin@dicp.ac.cn; lixianfeng@dicp.ac.cn

c Prof. Y. G. Wang
Department of Chemistry and Shanghai Key Laboratory of Molecular Catalysis and Innovative Materials, Institute of New Energy, iChEM (Collaborative Innovation Center of Chemistry for Energy Materials), Fudan University, Shanghai 200433, China

Experimental Section:

Materials:

$SnCl_4 \cdot 5H_2O$ (AR, 99 %, Aladdin and Macklin reagent), HBr (AR, Kermel Chemical Reagent Factory, Tianjin, China), HCl (AR, Kermel Chemical Reagent Factory, Tianjin, China), $SnBr_4$ (AR, 99 %, Aladdin), $SnBr_2$ (AR, 99 %, Aladdin), $SnCl_2 \cdot 2H_2O$ (AR, 98 %, Aladdin), $NiCl_2 \cdot 6H_2O$ (AR, 98 %, Kermel), choline chloride (ChCl, AR, Macklin, 98%), carbon felt (thickness was 5 mm, purchased from LIAOYANGJINGU), Nafion 115 (membrane) was obtained from Dupont.

Preparation of CNF/CNT-CF Electrode:

Firstly, pristine CFs (5 mm in thickness, 3 × 3 cm² in area) were pretreated via heat treatment at 550 °C in air for 5 h in a muffle furnace to increase the oxygen content. Subsequently, 2 g of nickel (II) chloride hexahydrate ($NiCl_2 \cdot 6H_2O$) was dissolved in 100 mL of ethanol. Eight pieces of the pretreated CFs were immersed into the $NiCl_2 \cdot 6H_2O$/ethanol solution, then stirring for 3 h and ultrasonic treatment for 30 min. Afterward, the soaked fibers were dried at 100 °C to obtain $NiCl_2$-loaded carbon fibers (denoted as $NiCl_2$@CFs).

Next, the as-prepared $NiCl_2$@CFs were calcined in an argon (Ar) atmosphere during a temperature-ramping stage until to 700 °C, then using a 10% H_2/Ar mixed gas (with a flow rate of 20 mL/min for H_2 and 200 mL/min for Ar) to reduce $NiCl_2$ to metallic nickel (Ni) for 2 h. Turned off the reducing gas, introducing a 10% acetylene (C_2H_2)/balanced Ar gas mixture (total flow rate: 380 mL/min) for 15 min. Finally, the sample was naturally cooled in an Ar atmosphere,

To remove the metallic Ni component, the above-synthesized sample was subjected to acid etching in 3 M sulfuric acid (H_2SO_4) for 24 h. After etching, the felt was rinsed with excess deionized water repeatedly until the pH of the rinse solution reached 7. The wet felt was then dried at 50 °C for 24 h, this sample was donated as CC-700.

For the preparation of CC-600 and CC-800, the same procedure as that for CC-700 were followed, except the temperature was raised to 600 °C (for CC-600) and 800 °C (for CC-800), respectively, then introducing the 10% C_2H_2/balanced Ar gas mixture for 15 min.

Electrochemical measurement:

The electrochemical performance of the different electrodes was evaluated using a typical three-electrode system, with all measurements were conducted on a Gamry Multichannel System (Model: Gamry Interface 1000). Reference electrode was Hg_2Cl_2/Hg (0.242 V vs. SHE), counter electrode was graphite plate (3 x 3 cm^2), the working electrode was round felt with 6 mm (diameter) and 2 mm (thickness). The cyclic voltammetry (CV) of anolyte was conducted in 20 mL solution that containing 20 mM $SnCl_4$ and 2 M HBr. CV tests at different scan rates (5~13 mV s^{-1}) were conducted in the electrolyte of 2 M HBr with 5 mM $SnBr_4$ and 5 mM $SnBr_2$. The EIS measurements were carried out in galvanostatic mode at a constant current of 5 mA, with a frequency range spanning from 100 kHz to 0.1 Hz

Materials characterization:

Scanning electron microscopy with EDS (SEM, JSM-7800F, JEOL) and transmission electron microscope (TEM, JEM-2100, JEOL) were used to characterize electrode morphology and the deposited tin layer. X-Ray Diffraction (XRD, D8 ADVANCE ECO; RIGAKU, Japan) was applied to characterize electrodes. Raman spectroscopy (Nano Wizard, manufacturer: Renishaw) was employed to characterize the coordination structures of stannic ions (Sn^{4+}), stannous ions (Sn^{2+}) and the structural characteristics of CC-T. Additionally, in-situ Raman spectroscopy was used to investigate the structural transformations during the valence state changes of tin. The specific

surface areas (SSAs) of the electrodes were tested via N_2 adsorption-desorption measurements, which were performed using a gas adsorption analyzer (ASAP-2010/ASAP 2010M), the data were calculated from the adsorption branches of the isotherms using the Brunauer-Emmett-Teller (BET) method. X-ray photoelectron spectroscopy (XPS, Thermofisher Escalab 250 Xi+) was employed to characterize the surface chemical composition, chemical bonding states of C and O elements, and surface oxygen content of the electrode material. Inductively Coupled Plasma Optical Emission Spectroscopy (ICP-OES, PerkinElmer, Avio 550 Max) was employed to determine the concentration of tin ions.

Density functional theory (DFT) calculations:

All structural optimizations and energy calculations in this work were conducted using the Vienna ab initio simulation package (VASP6.4.2) based on the spin-polarized density functional theory (DFT)[55]. For the generation of pseudopotential, the projected augmented wave method with a cutoff energy of 450 eV and Perdew−Burke−Erzenh functional in the generalized gradient approximation (GGA) was employed[56]. The empirical revision of Grimme's scheme take into account using the van der Waals interaction (DFT−D3) [57, 58]. The carbon nanotube (CNT) model was built using Material Studio software. The carbon nanotube had a diameter of 4.07 angstroms and a length of five structural units. For CNT@OH/COOH, we connected two pairs of OH and COOH groups on the C ends of CNT. The Gamma point was used for the catalysts. All the structural models were optimized to the ground state until the convergence of energy and the residual forces less than 10^{-5} eV and 0.02 eV $Å^{-1}$, respectively. The VASPKIT code was used for postprocessing computational data obtained from VASP[59].

Sn-Br battery test:

A Sn-Br flow battery was assembled by sandwiching a Nafion 115 membrane (DuPont, USA) between a positive electrode (PCF) and a negative electrode (CC-T or PCF). The assembly was maintained at a compression ratio of approximately 0.8, with an effective electrode area of 3 × 3 cm^2, and two polar plates were used to clamp the electrode-membrane assembly.

Battery performance tests were conducted using a Neware battery test system (5 V, 12 A; Neware Corp., Shenzhen, China). For 1 M electrolytes, the state-of-charge (SOC) was approximately 70%, the current densities of charge-discharge tests were 40 mA cm^{-2}, and the discharge cutoff voltage was set at 0.4 V, the common electrolyte was composed of 1 M $SnCl_4{\cdot}5H_2O$, 2 M HBr, and 0.16 M choline chloride (ChCl; used as a complexant to inhibit bromine diffusion). The negative electrolyte (8 mL) was delivered via a peristaltic pump at a set flow rate of 60 mL min^{-1}, while the positive electrolyte (50 mL) was circulated using a magnetic pump. For 2 M electrolytes, anolyte: 2 M $SnCl_4$ + 0.16 M ChCl + 2 M HBr, 8 ml; catholyte: 1 M $SnCl_4$ + 0.16 M ChCl + 2 M HBr, 100ml, the battery was charged to 1.5 V and subsequently discharged to 0.4 V. For 4 M electrolytes, anolyte: 4 M $SnCl_4$ + 0.16 M ChCl + 2 M HBr, 8 ml; catholyte: 1 M $SnCl_4$ + 0.16 M ChCl + 2 M HBr, 100ml, the battery was charged to 1.5 V and subsequently discharged to 0.4 V. The battery was tested at current densities ranging from 40 to 120 mA cm^{-2}, charging to 70% SOC and discharging to 0.4 V, the electrolyte was same as the above 1 M electrolytes. The battery of high specific capacity was assembled same as the above 1 M electrolytes, except the battery was charged to 1.5 V. The high areal capacity Sn-based battery: adopted the aforementioned 1 M common electrolyte, with 60 mL for the anode and 300 mL for the cathode, the battery was charged to 1.5 V and subsequently discharged to 0.4 V. All electrolytes in the experiments were prepared using ultra-pure water.

In-Situ Raman Spectroscopy

The in-situ evaluation system enabled simultaneous flow battery charge-discharge operation and Raman detection. A transparent quartz tube was installed in the pipeline on the anode side of the flow battery, positioned beneath the Raman detector for signal collection. The electrolyte configurations used as follows: 1 M $SnCl_4$ + 0.16 M ChCl + 2 M HBr (12 ml for the anolyte, 50 ml for the catholyte). Test was conducted under constant current conditions of 80 mA cm^{-2}, with charging to 1.7 V and discharging to 0.4 V. The exposure time was set to 60 seconds, with laser intensity at 5% and integration times was 1.

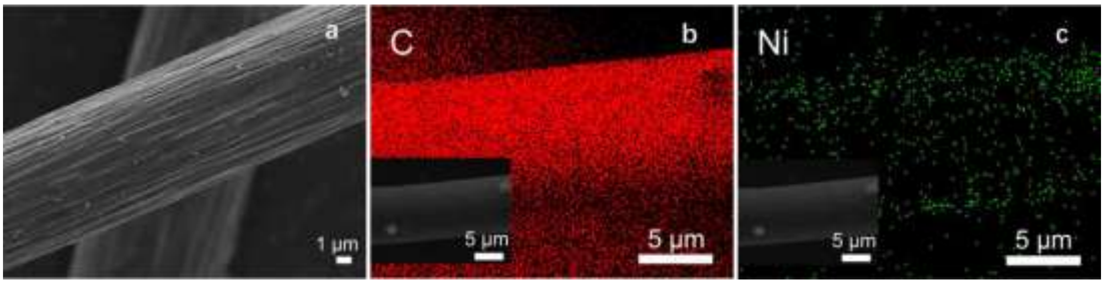

Figure S1. **(a)** SEM image of the $NiCl_2$-treated CF: **(b)** EDS mapping of C and **(c)** Ni. The insets in **(b)** and **(c)** correspond to the SEM images of the respective regions.

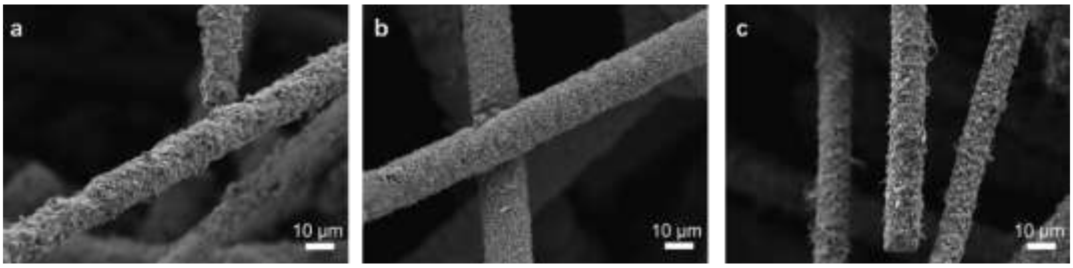

Figure S2. SEM images of **(a)** CC-600, **(b)** CC-700 and **(c)** CC-800. In-situ grown carbon nanotubes (CNTs) are uniformly deposited on the surface of CF.

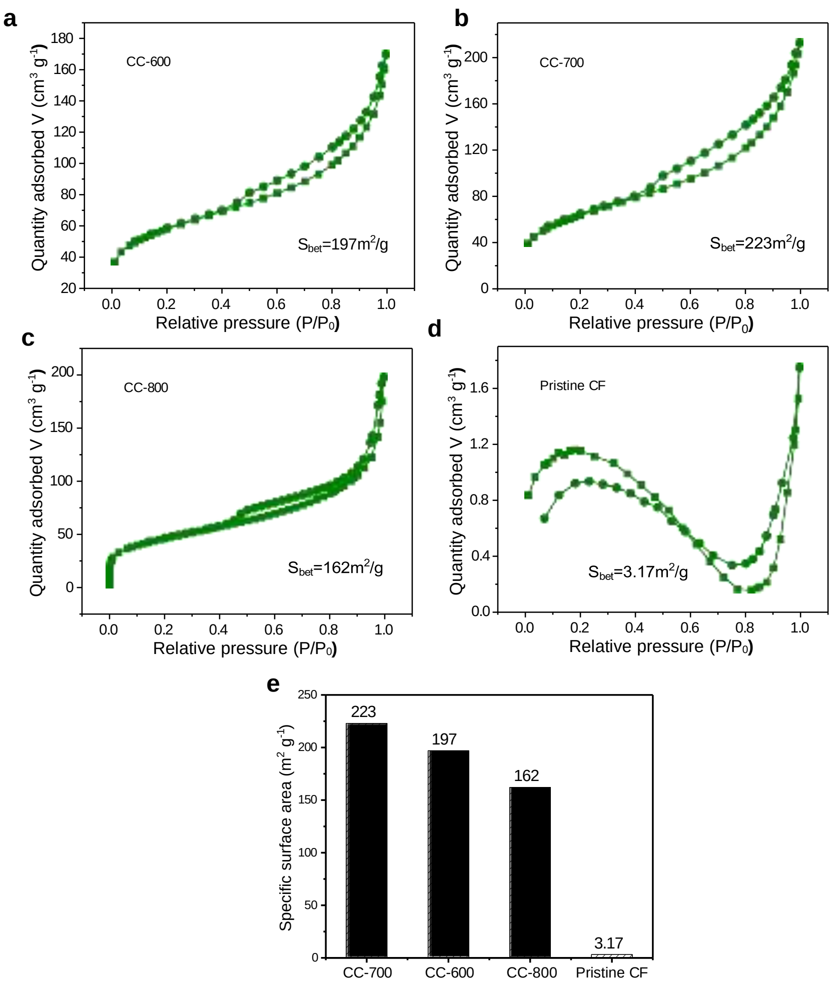

Figure S3. Adsorption and desorption isotherms of **(a)** CC-600, **(b)** CC-700, **(c)** CC-800, and **(d)** pristine CF. **(e)** Comparison of the specific surface area across different electrode materials.

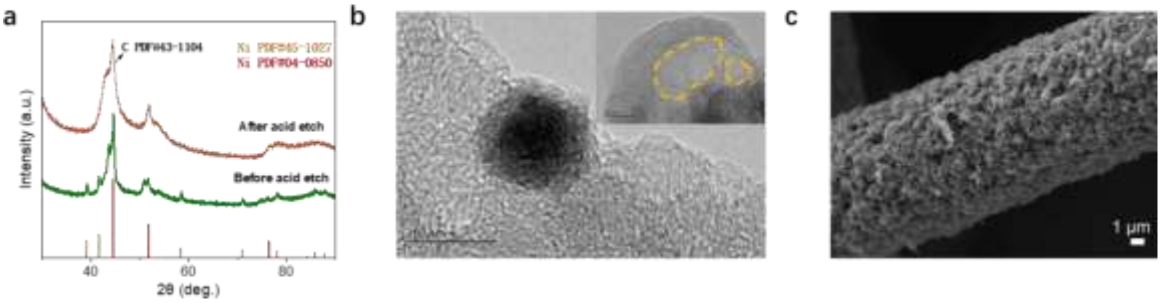

Figure S4. (a) XRD comparison patterns of CC-700 before and after acid etch. **(b)** TEM images of CC-700 before and after acid etch (inset). **(c)** SEM images of CC-700 after acid etch. The results indicate that the morphology of the CC-700 remains unchanged after etching.

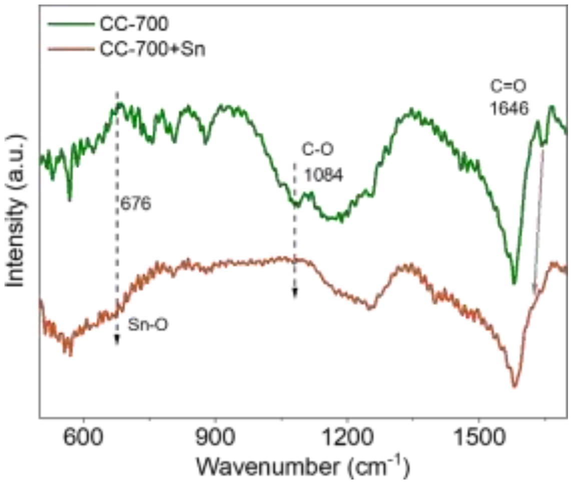

Figure S5. FT-IR spectra of CC-700 and CC-700+Sn ions electrodes.

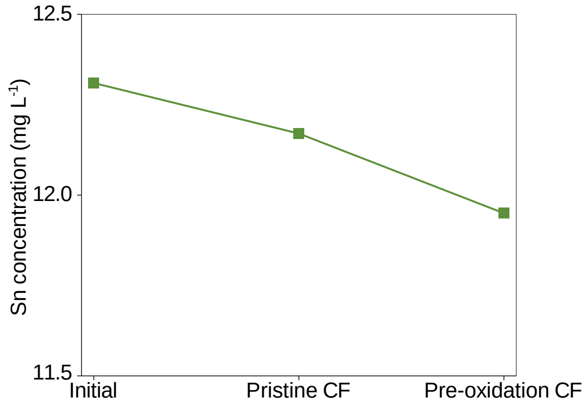

Fig S6. Comparison curve of the remaining Sn ion after adsorption test using pristine CF vs. pre-oxidized CF.

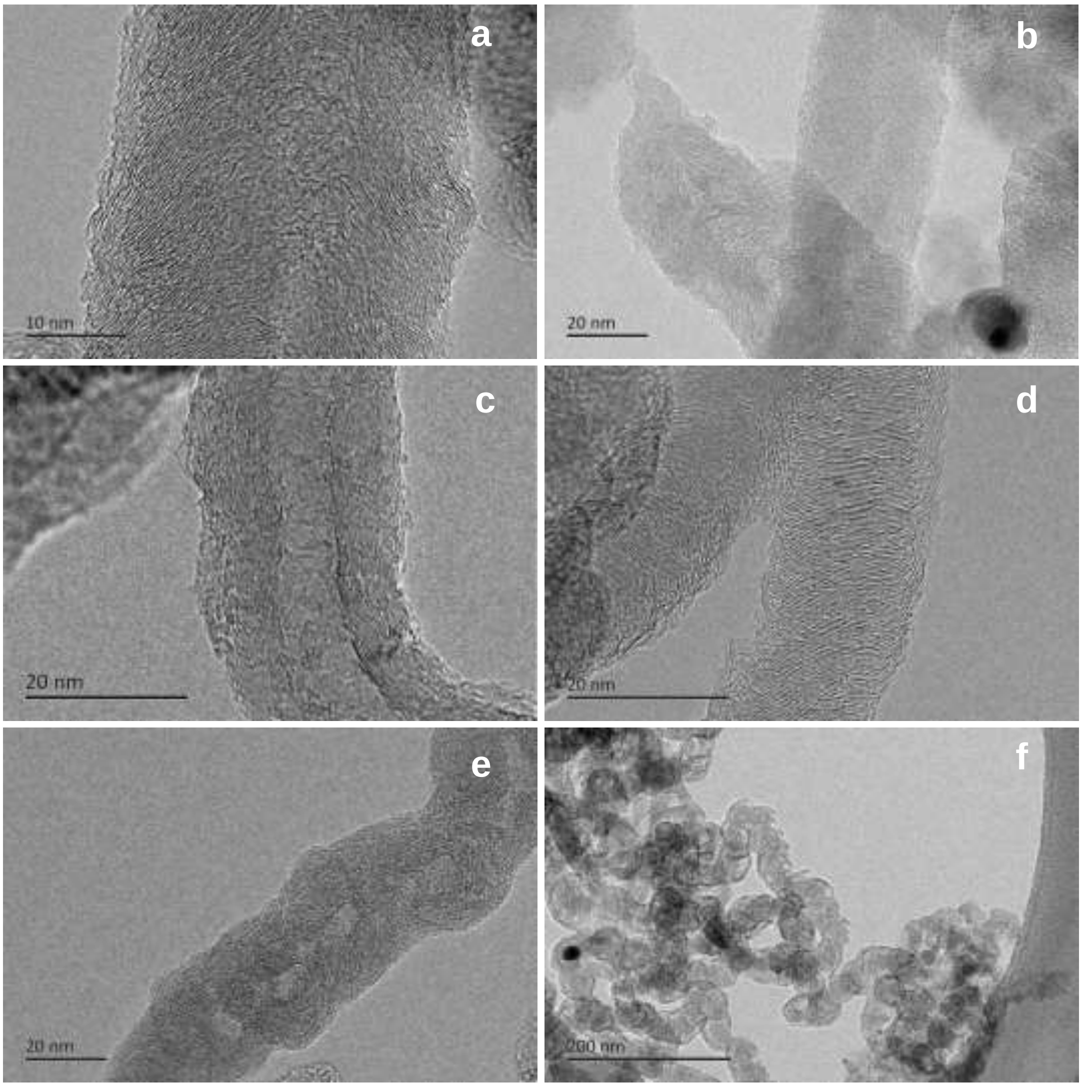

Figure S7. TEM images of **(a, b)** CC-600. **(c, d)** CC-700. and **(e, f)** CC-800.

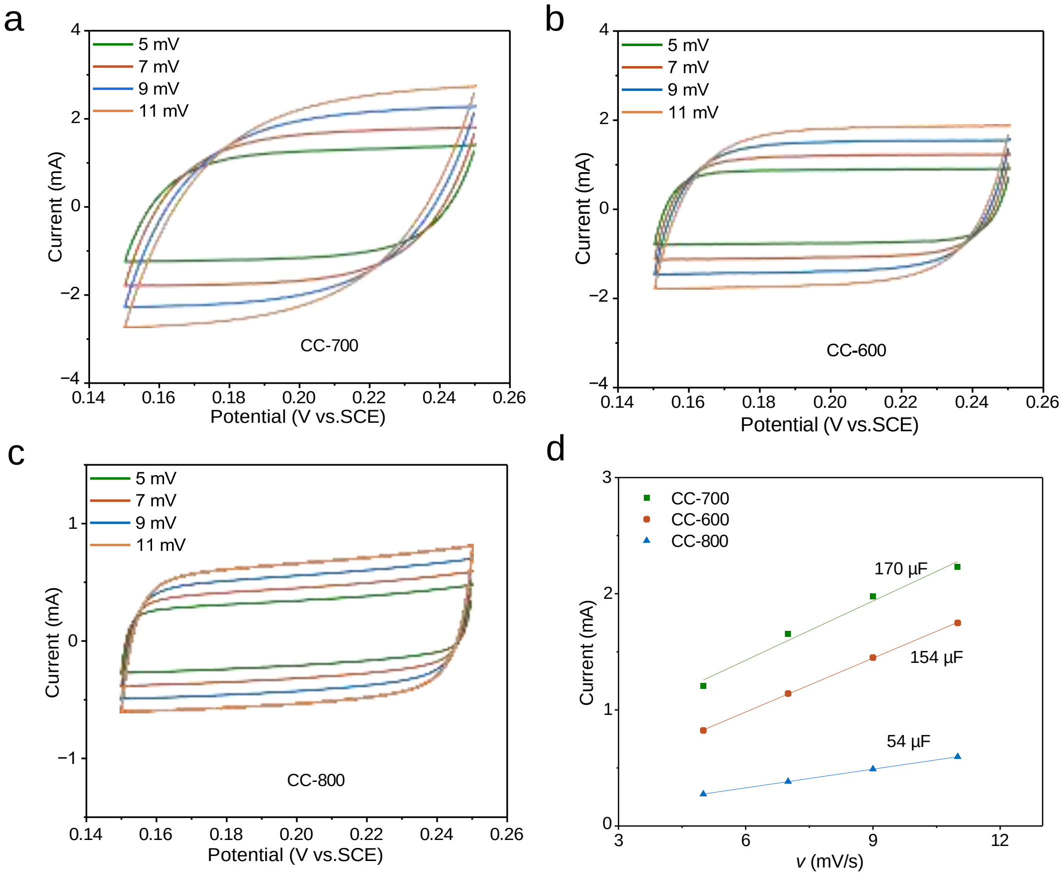

Figure S8. CV curves of the electrodes at different scan rates. (a) CC-700. **(b)** CC-600. **(c)** CC-800. **(d)** The current and sweep rate exhibit a linear relationship, the current at this stage was capacitive current stem from the potential at 0.2 V, the slope value is Cdl, the double layer capacitance. The tests were conducted at the voltage near to open-circuit voltage (OCV).

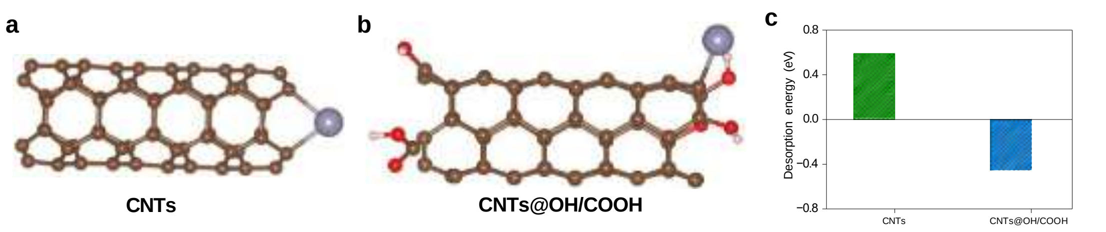

Figure S9. Desorption model diagrams for Sn atom on (a) CNTs and (b) CNTs@OH/COOH. (c) Desorption energy simulation of Sn atom on CNTs and CNTs@OH/COOH, respectively.

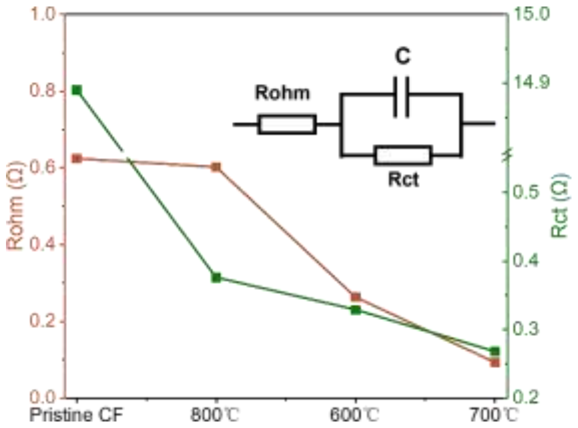

Figure S10. The equivalent circuit and associated resistances, including Rohm (ohmic resistance) and Rct (charge transfer resistance). The electrolyte is composed of 1 M $SnCl_4$+2 M HBr+0.16 M ChCl. Before testing, the batteries were charged to a state of charge (SOC) of 25%.

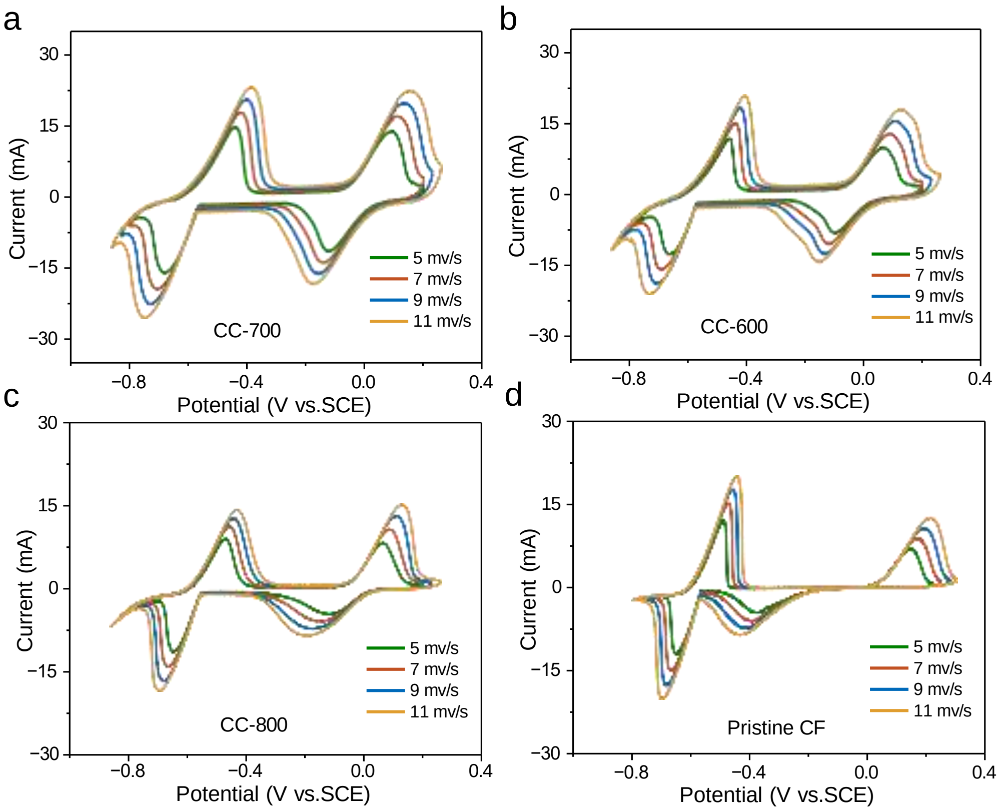

Figure S11. Cyclic voltammetry (CV) results at various scan rates using **(a)** CC-700, **(b)** CC-600, **(c)** CC-800, and **(d)** pristine CF as working electrodes. The pristine CF showed the largest peak potential difference of Sn^{4+}/Sn^{2+} with the poorest reversibility. The experiments were conducted in an electrolyte comprising 2 M HBr + 5 mM $SnBr_4$ + 5 mM $SnBr_2$.

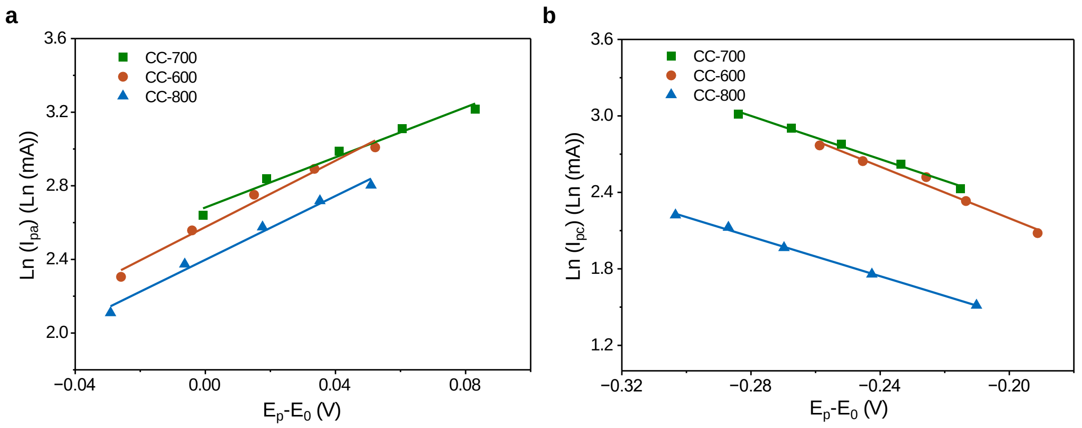

Figure S12. By referring to the peak current and peak potential of the Sn^{2+}/Sn^{4+} reaction with different scan rates in Figure 10, obtain the fitted linear curve of ln($I_{pa/pc}$) versus E_p-E_0 with different electrodes, where (a) corresponds to the oxidation process of Sn^{2+} and (b) to the reduction process of Sn^{4+}.

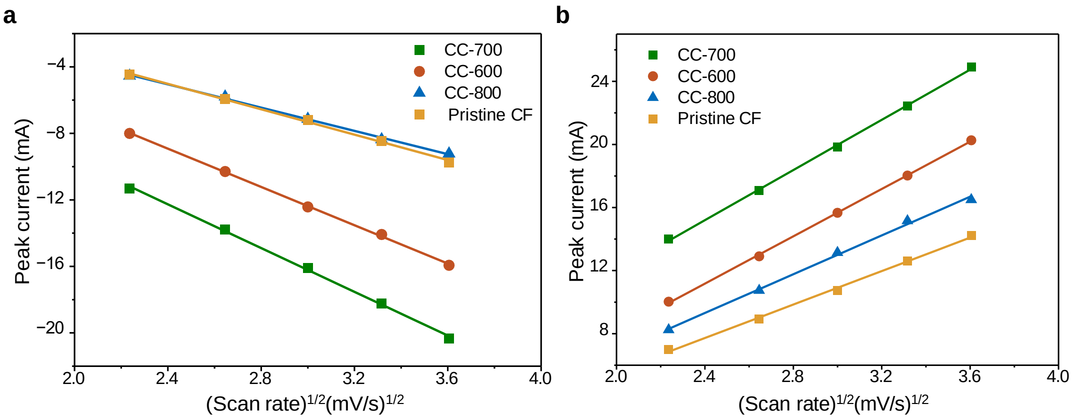

Figure S13. By referring to the peak current of the Sn^{2+}/Sn^{4+} reaction with different scan rates in Figure 11, obtain the fitted linear curve of $I_{pa}/_{pc}$ versus $v^{1/2}$ with different electrodes, where (a) corresponds to the reduction process of Sn^{4+} and (b) to the oxidation process of Sn^{2+}. For the calculation of the apparent diffusion coefficient D_{app}, refer to Table S2.

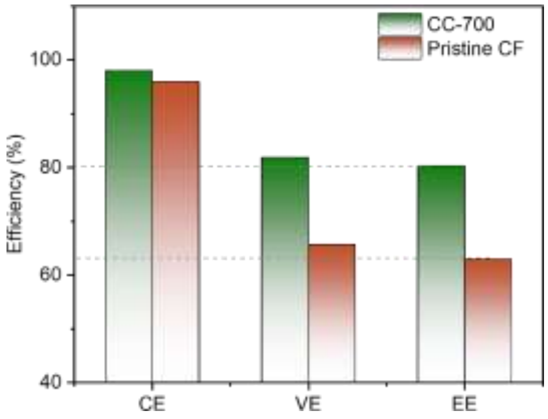

Figure S14. Battery efficiencies of different electrodes at 40 mA cm^{-2}.

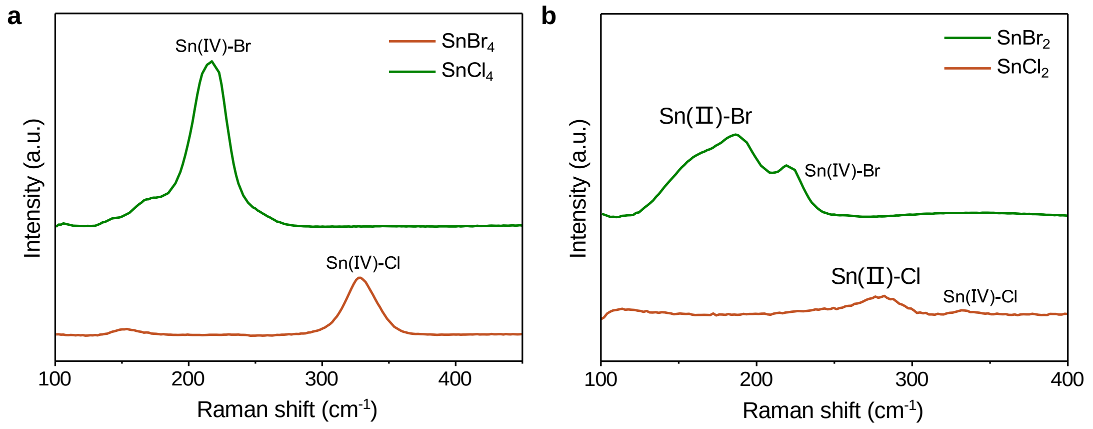

Figure S15. Raman spectra of the characteristic signal of Sn^{4+}-X **(a)** and Sn^{2+}-X **(b)** (with X = Cl^-, Br^-).

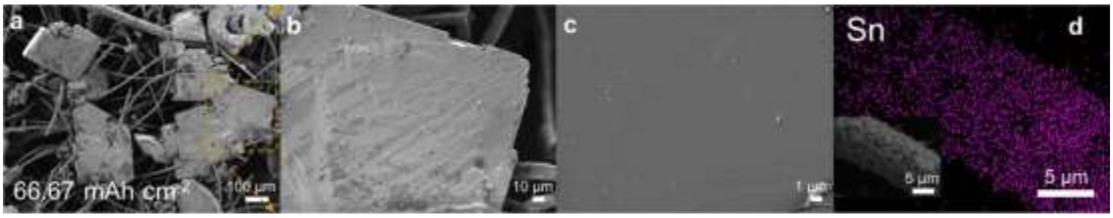

Figure S16. (a-c) SEM images of Sn deposition. **(d)** EDS mapping of Sn, with the inset in **(d)** corresponding to the SEM image.

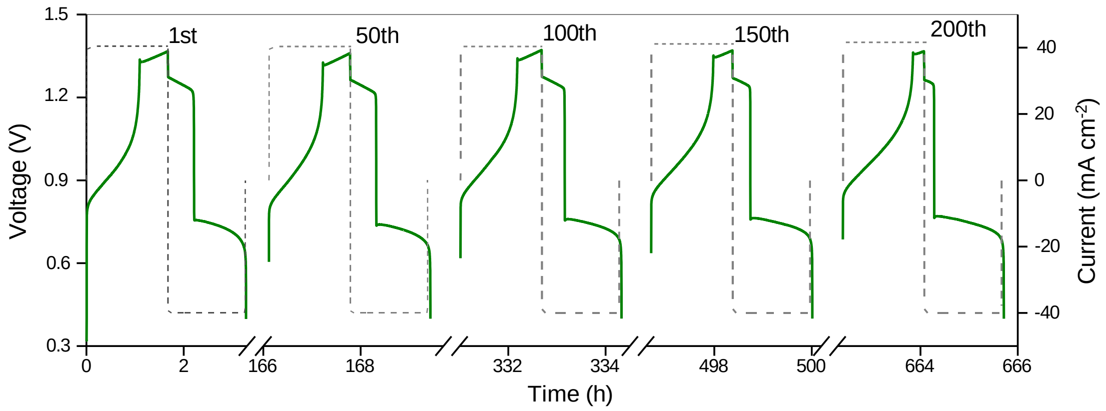

Figure S17. The voltage curves of Sn-Br flow batteries (from **Figure. 4d**) at current of 40 mA cm^{-2}.

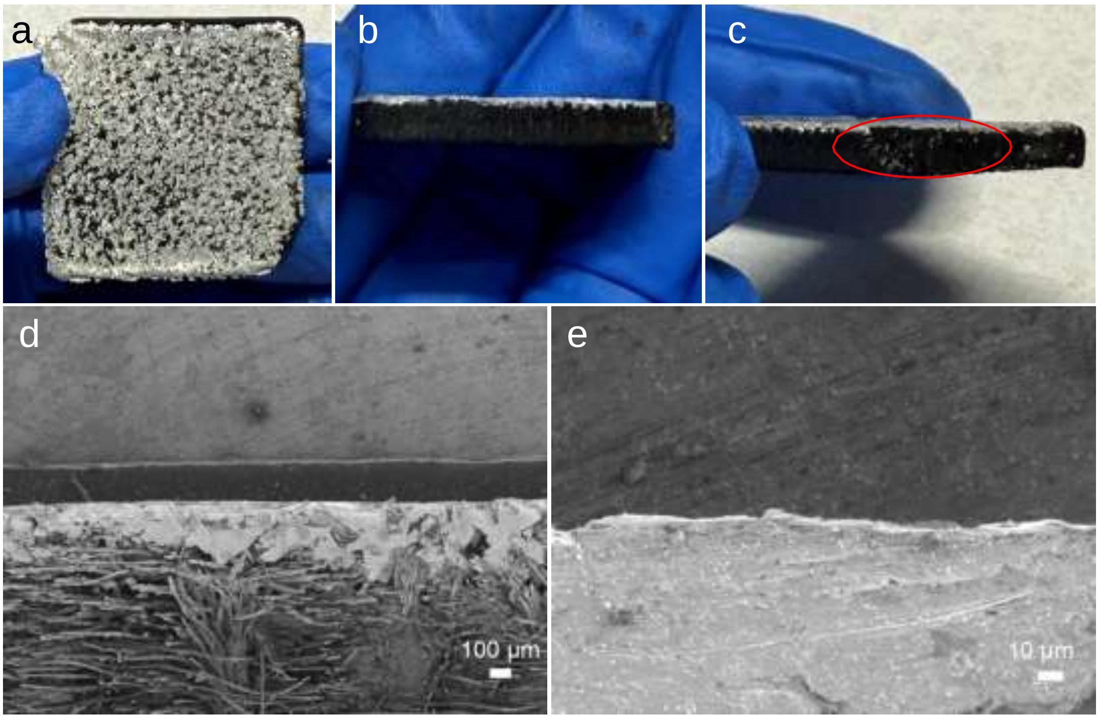

Figure S18. Morphological characterization of Sn deposits after long-term cycling. (a-c) Photographs of CC-700 after Sn deposition. (d-e) SEM images of the cross-sectional micrograph of Sn deposits.

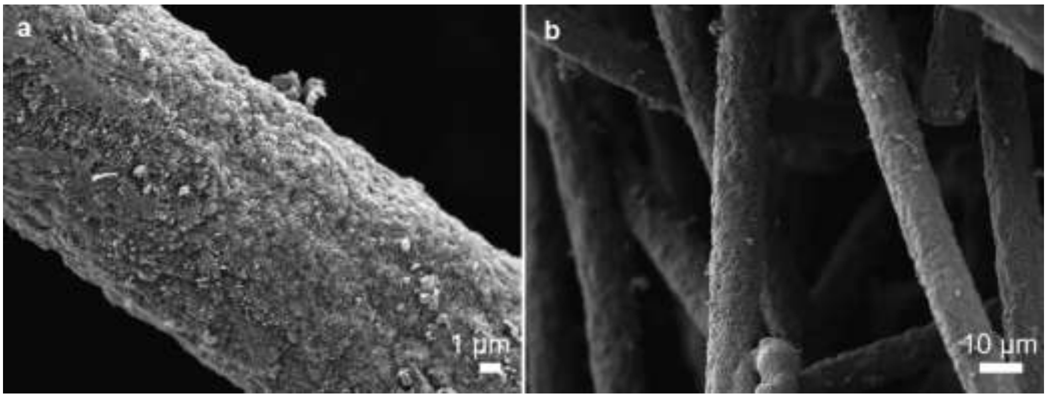

Figure S19. SEM images of CC-700 after cycling test, derived from **Figure 4d**.

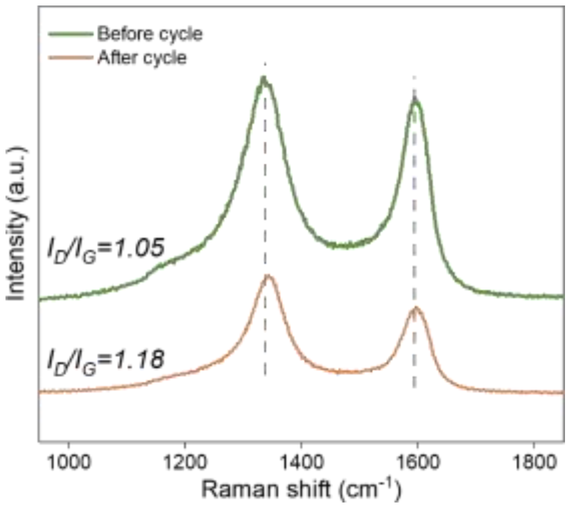

Figure S20. Raman spectra of the CC-700 before and after long-cycling test.

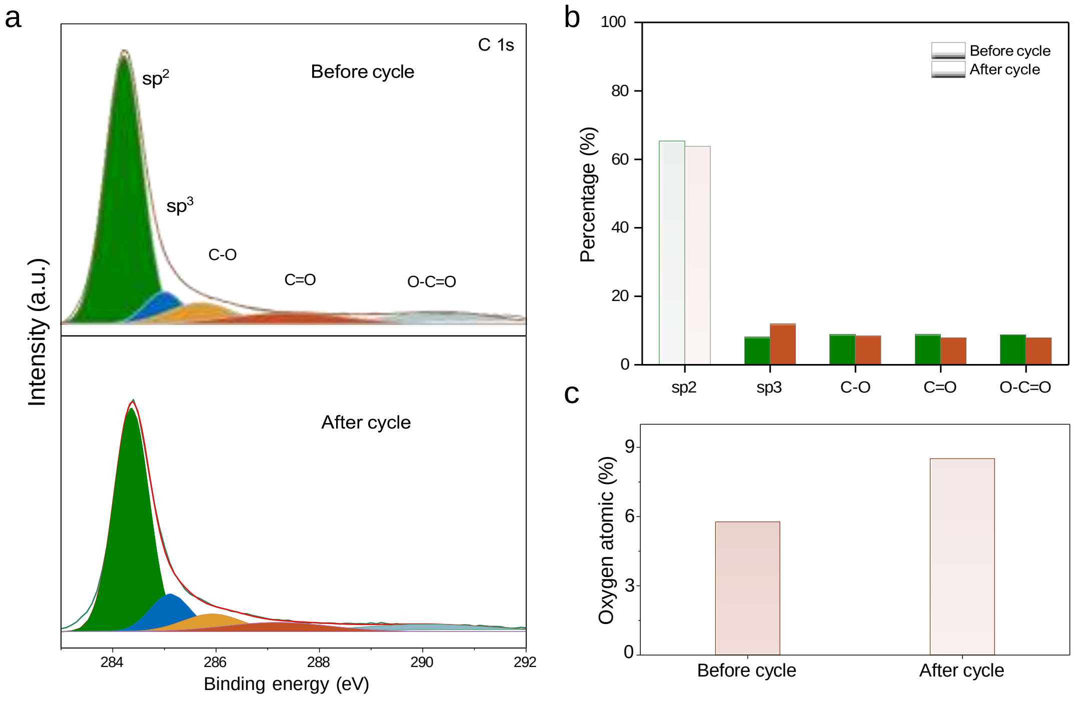

Figure S21. XPS analysis of CC-700 before and after long-cycling test. (a) High-resolution spectra of C 1s peaks. (b) Ratios of functional groups calculated from the XPS results in (a). (c) Comparison of the oxygen content.

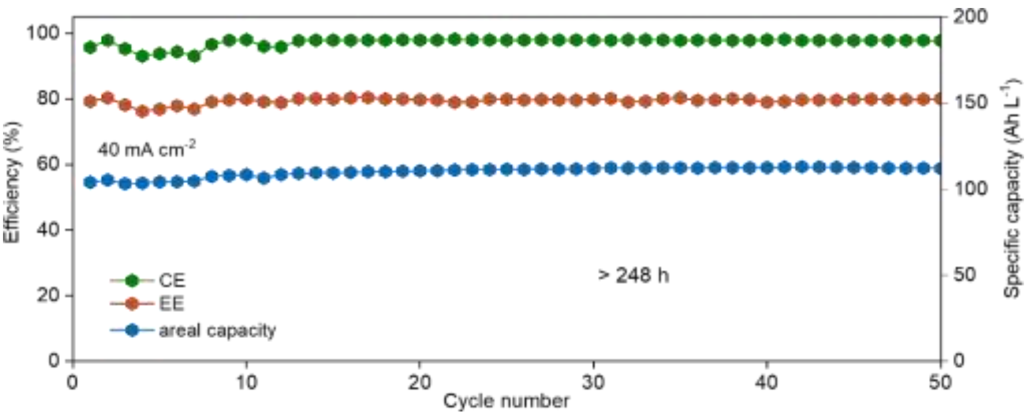

Figure S22. Long-term performance of the battery with high electrolyte utilization. Electrolyte:1 M $SnCl_4$ +2 M HBr+ 0.16 M ChCl.

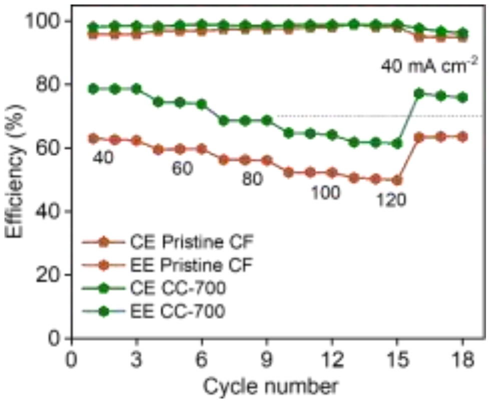

Figure S23. The batteries efficiencies of pristine CF and CC-700, the currents densities are range from 40 to 120 mA cm^{-2}.

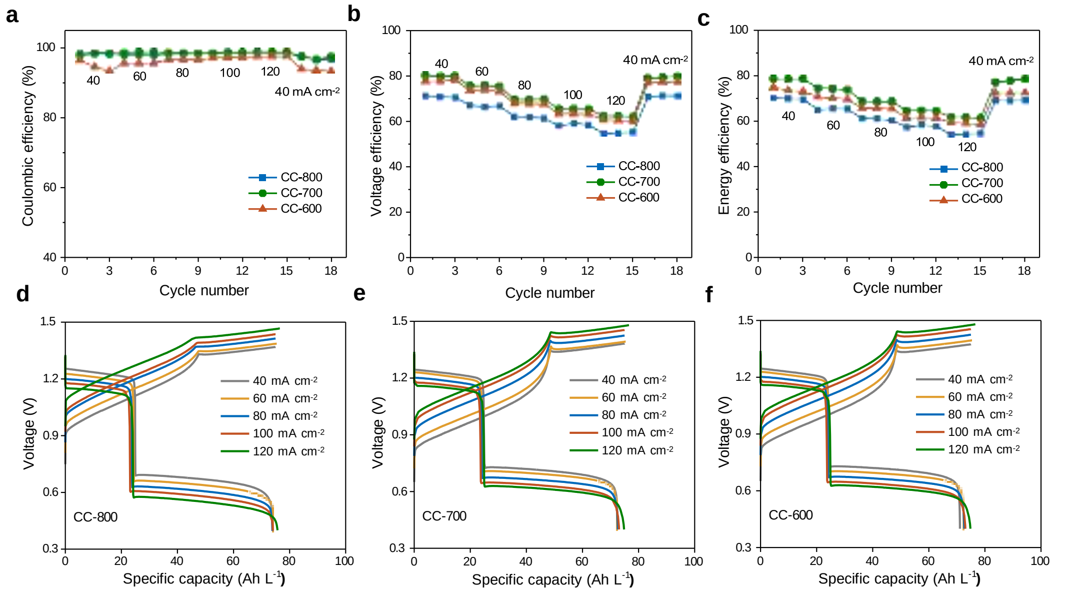

Figure S24. The battery efficiencies with different electrodes, with current densities ranging from 40 to 120 mA cm^{-2}. (a) CE, **(b)** VE, **(c)** EE. And the corresponding charge-discharge curves with different electrodes, **(d)** CC-800, **(e)** CC-700, **(f)** CC-600.

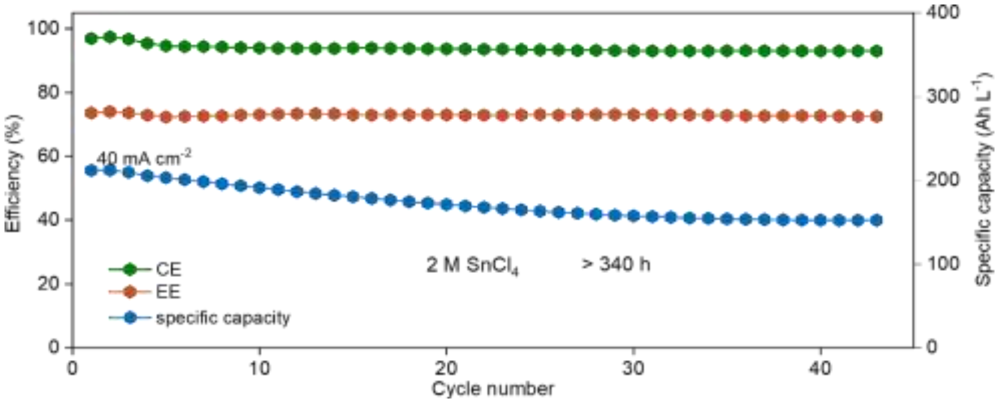

Figure S25. Long-term performance of the battery with anolyte containing 2 M $SnCl_4$. Anolyte: 2 M $SnCl_4$ + 0.16 M ChCl + 2 M HBr; Catholyte: 1 M $SnCl_4$ + 0.16 M ChCl + 2 M HBr.

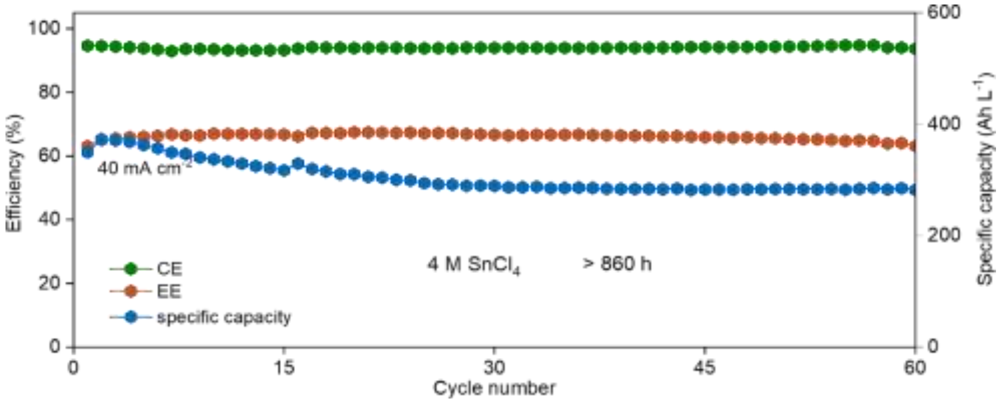

Figure S26. Long-cycle performance test of the battery with high concentration of $SnCl_4$ (4 M). Anolyte: 4 M $SnCl_4$ + 0.16 M ChCl + 2 M HBr; Catholyte: 1 M $SnCl_4$ + 0.16 M ChCl + 2 M HBr.

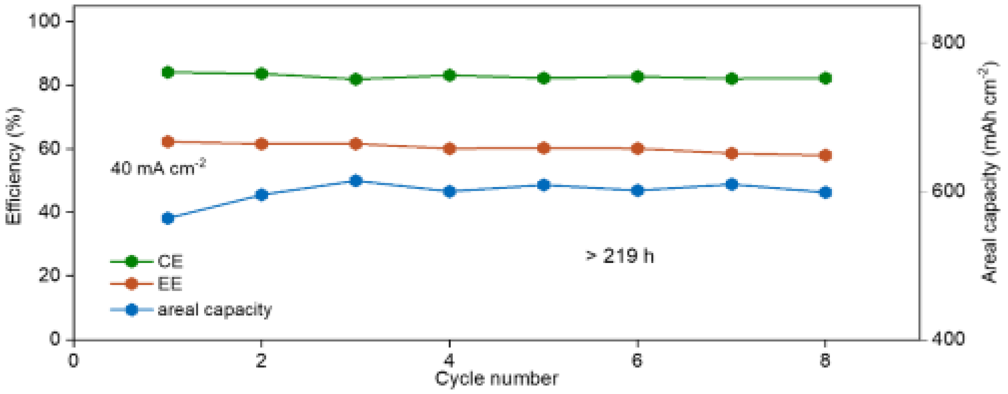

Figure S27. Long-cycle performance test of the battery with high areal capacity of 600 mAh cm^{-2}.

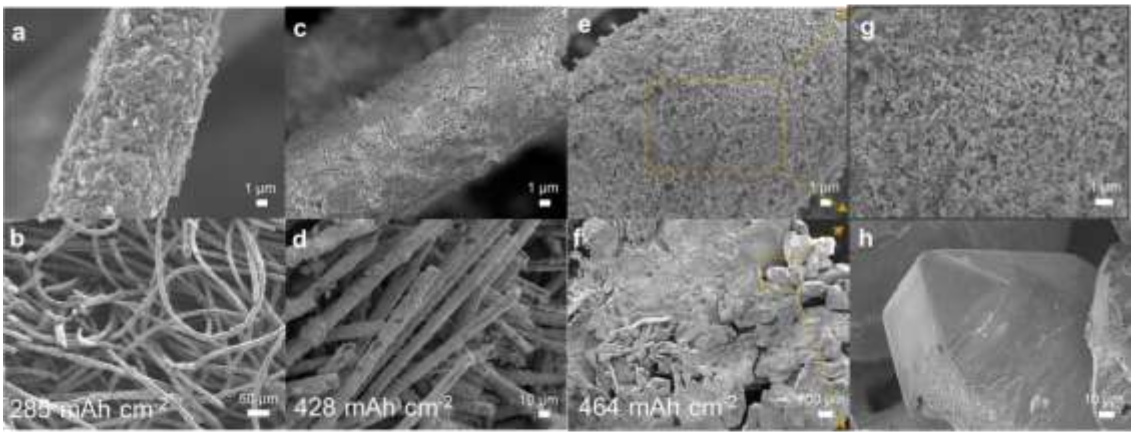

Figure S28. SEM images of tin deposition at varying areal capacities. **(a-b)** Areal capacity of 285 mAh cm^{-2}, **(c-d)** 428 mAh cm^{-2}, and **(e-h)** 464 mAh cm^{-2}. The tin deposition morphology remains uniformly smooth, with no dendrite formation observed across all tested areal capacities.

Table S1. The intrinsic rate constant k_0 in various electrodes

Electrolyte	ECSA (cm^2)	$Sn^{4+} \rightarrow Sn^{2+}$		$Sn^{2+} \rightarrow Sn^{4+}$	
		$Interceptd$	k_0 ($cm\ s^{-1}$) x 10^{-2}	$Interceptd$	k_0 ($cm\ s^{-1}$) x 10^{-2}
CC-600	1.93	0.17	0.14	2.58	1.56
CC-700	2.05	0.63	0.20	2.68	1.57
CC-800	0.68	-0.11	0.30	2.40	3.70

$$I_P = 0.227 nFACk^0 \exp\left[-\frac{\alpha nF}{RT}(E_p - E^0)\right]$$

E_p : anode peak potential (V); E^0 = -0.0915 V (vs SCE); F : faraday constant, 96485 C/mol;
C: concentration of $SnCl_4$ ($10*10^{-3}$ mol/L);
α: charge transfer coefficient (0.5); A: the surface of electrode (ECSA is for calculating k_0; the geometric area (0.28274 cm^2) is for k_{app})

Table S2. The diffusion coefficient D_{app} in various electrodes

Electrode	$Sn^{4+} \rightarrow Sn^{2+}$		$Sn^{2+} \rightarrow Sn^{4+}$	
	Slope k	D_{app} ($cm^2 s^{-1}$) x 10^{-6}	*Slope k*	D_{app} ($cm^2 s^{-1}$) x 10^{-6}
CC-600	-5.76	7.17	7.50	12.15
CC-700	-6.58	9.36	7.96	13.69
CC-800	-3.50	2.65	6.15	8.17
PCF	-3.82	3.15	5.31	6.09

$$I_P = 2.69 \times 10^5 n^{3/2} A D_O^{1/2} v^{1/2} C$$

I_P : peak current（mA); n: number of electron transfer (n=2);

C: concentration of Sn ($10*10^{-3}$ mol/L); D_{app}: diffusion coefficient;

A: the surface of electrode, 0.28274 cm^2; v: scan rate

References:

55. J. Hafner, *J. Comput. Chem.*, 2008, **29**, 2044-2078.
56. G. Kresse and D. Joubert, *Phys. Rev. B*, 1999, **59**, 1758-1775.
57. K. B. J. P. Perdew, M. Ernzerhof, *J. Comput. Chem.*, 1996, **77**, 3865-3868.
58. S. Grimme, *J. Comput. Chem.*, 2006, **27**, 1787-1799.
59. V. Wang, N. Xu, J. C. Liu, G. Tang and W. T. Geng, *Comput. Phys. Commun.*, 2021, **267**, 19.
